# The effect of multifrequency ultrasound treatments on structure, rheological, and digestive properties of starch in frozen wheat dough

**DOI:** 10.1016/j.ultsonch.2025.107507

**Published:** 2025-08-25

**Authors:** Zahoor Ahmed, Abid Hussain, Umar Farooq, Yibin Zhou, Isam A. Mohamed Ahmed, Muhammad Waseem, Bin Xu, Muhammad Faisal Manzoor, Robert Mugabi

**Affiliations:** a School of Food and Nutrition, Anhui Agricultural University, Hefei 230036, China; b School of Food and Biological Engineering, Jiangsu University, Zhenjiang 212013, China; c Department of Agriculture & Food Technology, Karakoram International University, Gilgit, Baltistan, Pakistan; d Department of Nutrition and Dietetics, National University of Medical Sciences Rawalpindi, Pakistan; e Department of Food Sciences and Nutrition, College of Food and Agricultural Sciences, King Saud University, P.O. Box 2460, Riyadh 11451, Saudi Arabia; f Department of Food Science and Technology, Faculty of Agriculture and Environment, Islamia University of Bahawalpur, 63100, Pakistan; g School of Food Science and Engineering, Foshan University, Foshan, China; h Faculty of Sciences and Technology, ILMA University, Karachi, Pakistan; i Department of Food Technology and Nutrition, Makerere University, Kampala, Uganda

**Keywords:** Ultrasound treatment, Wheat frozen dough, In vitro digestibility, Functional properties, Rheological, Structure analysis

## Abstract

This study explores the impact of multifrequency ultrasonic treatments on the properties of wheat frozen dough starch at different treatment times (15, 25, and 35 min). Fundamental properties analyzed included particle size, solubility, swelling power, differential scanning calorimetry (DSC), X-ray diffraction (XRD), Fourier-transform infrared spectroscopy (FTIR), morphological traits, digestibility, and rheological characteristics. Results indicate that ultrasonication (US) enhances the amylose content, solubility, and swelling power of the frozen wheat dough starch. Treatment led to a significant increase (p < 0.05) in rapidly digestible starch (RDS) and resistant starch (RS) with a longer exposure time. Specifically, RDS content rose 30 % with multifrequency ultrasound (T2 20/40/60/25 min), while RS and slowly digested starch (SDS) decreased at 35 min. FTIR analysis revealed notable peaks between 3290 and 3299 cm^−1^, corresponding to the O-H stretching of hydroxyl groups. Rheological characteristics increased in storage modulus (G’) and loss modulus (G’’) after 25 min (20/40/60) of US, then decreased at 35 min. Ultrasound treatment caused surface depressions and pores on wheat starch granules in the frozen dough. This research highlights the efficacy of multifrequency US in augmenting the functional, rheological, and ultra-structural properties of wheat frozen dough. By counteracting the deleterious effects of freezing, this innovative approach shows considerable promise in enhancing the processing quality of frozen dough starch.

## 1 Introduction

Wheat is a staple ingredient in numerous food products due to its unique functional properties, which include its ability to form a viscoelastic dough, which is essential for bread making and baked goods [1]. Achieving the optimal combination of nutritional, functional, rheological, and structural characteristics in wheat-based products remains challenging in food science [2]. It is due to the complex interplay of wheat’s inherent properties, such as starch content and protein matrix, which are not always conducive to meeting consumer expectations for texture, shelf life, and nutritional quality [3]. Frozen wheat dough has become a staple in the food industry, particularly for its convenience and extended shelf life [4]. Despite these advantages, maintaining the quality and functionality of frozen dough presents significant challenges. The freezing and thawing processes often lead to a reduction in dough elasticity, decreased gas retention, and changes in the dough’s rheological properties [5]. These issues can result in a final product with inferior texture, volume, and overall sensory qualities, impacting consumer satisfaction [6]. Traditional methods of improving frozen dough have included chemical additives and enzymatic treatments, but these approaches often come with drawbacks, including potential health concerns and regulatory limitations.

Over the years, various modification techniques have been employed to enhance the functionality. These include chemical, genetic, and physical modification, such as ultrasound US, each with distinct advantages and limitations [7]. US treatment gained prominence in the food industry due to its ability to enhance product quality while minimizing the use of chemical agents. The US offers a promising approach to modifying the properties of wheat starch in a controlled and environmentally friendly manner [8].

The primary mechanism by which the US modifies wheat starch is cavitation, a process where gas bubbles rapidly form and collapse in the medium, generating intense shear forces. These forces disrupt the amorphous regions of the wheat starch granules, increasing their surface area by creating pores and fissures. Unlike traditional methods such as acid hydrolysis, US achieves these modifications without altering the overall size and shape of the granules, thus preserving the starch’s structural integrity and enhancing its physicochemical properties [9]. Applying multifrequency US to wheat frozen dough starch offers several advantages over traditional methods. Ultrasound-assisted freezing can improve the textural properties of dough and reduce the damage to gluten structure, potentially affecting the overall digestibility of the dough [10]. The combination of freezing and ultrasound treatment can lead to a more uniform and smaller network structure similar to fresh protein, which may influence the digestibility of the starch in the dough.

It is a non-thermal, environmentally friendly process that reduces the need for chemical additives, making it an attractive option for clean-label food products. The US processing can be precisely controlled, allowing the fine-tuning of dough properties to meet specific product requirements. Future research may optimize US parameters to achieve the desired dough characteristics while minimizing energy consumption [11]. Multifrequency US presents a novel and practical approach to improving wheat frozen dough’s nutritional, functional, rheological, and structural characteristics. By addressing the inherent challenges of frozen dough, this technology can revolutionize the production of high-quality, convenient bakery products [12].

This study aims to bridge critical gaps in understanding the role of multifrequency US in enhancing the quality of wheat frozen dough by comprehensively evaluating its impact on key physiochemical functional and structural properties by leveraging advanced techniques such as rheological analysis Differential scanning calorimetry DSC X-ray diffraction XRD Fourier-transform infrared spectroscopy (FTIR) and in vitro digestibility assays. These modifications are expected to improve frozen dough rheological behavior, thermal stability, and morphological integrity, offering valuable insights into optimizing US parameters for industrial applications. Ultimately, this study seeks to establish the multi-frequency US as a pioneering and sustainable tool for enhancing the quality, functionality, and nutritional value of wheat-based frozen products, contributing to advancements in non-thermal food processing technologies.

## 2 Materials and methods

### 2.1 Materials

Wheat flour was obtained from Wudeli Flour Group Co Ltd, Handan, China. Its proximate composition was as follows on a wet weight basis: moisture 12.5 % protein 16.0 % fat 3.0 %, ash 1.5 % fiber 1.0 % and carbohydrates by difference 66.0 %. All chemicals and reagents used in the analysis were of analytical grade.

### 2.2 Dough samples preparation

Dough samples were prepared following the method outlined by [13], using a formulation of 500 g of wheat flour, 4 g of salt, and 295 g of deionized water. The ingredients were mixed for 22 min using an AM-CG108 mixer (North American Electric, Zhuhai, China) and proofed at 30 °C with 80 % relative humidity for 30 min in an XF-16A proofer (Hongling Electric Heating Equipment, Guangzhou, China). After proofing, the dough was portioned into 50-gram pieces, sealed in self-sealing bags, and subjected to multifrequency ultrasound treatment.

### 2.3 Ultrasound (US) treatment

Fifty grams of dough were pretreated in a WKS1800/7S ultrasonic (US) reaction tank (Jiangsu, China). for pretreatment. Three US frequency modes were tested: single-frequency 20 kHz, dual-frequency 20/40 kHz, and triple-frequency 20/40/60 kHz. Treatment durations were set at 15, 25, and 35 min (as detailed in Table 1). A power density of 65 W/L was consistently maintained, with the temperature during treatment controlled between 2–8 °C. In the following US treatment, the samples were stored at −80 °C for seven days and subsequently dried using a freeze-dryer. The control sample underwent the same process without US treatment.

**Table 1:** The treatment plan, particle size, and amylose content of frozen dough were treated with different multi-frequency ultrasonic treatments.

Sample code	Treatment plan			D[3,4] (µm)	D_[0.1]_ (µm)	D_[0.5]_ (µm)	Span Index	Amylose Content (%)
	Frequency (kHz)	Time (min)	Power (W/L)					
Control	NA	NA	65	60.2 ± 0.9 ^a^	211 ± 2^a^	177 ± 2^a^	0.065 ± 0.001^e^	19.31 ± 054^a^
S1	20	15	65	55.8 ± 1.3^b^	208 ± 3^a^	172 ± 2^b^	0.075 ± 0.002^d^	20.58 ± 0.91^b^
S2	20	25	65	54.4 ± 2.8^b^	206 ± 2^a^	167 ± 2^c^	0.077 ± 0.004^d^	22.09 ± 0.43c
S3	20	35	65	52.4 ± 2.8^c^	204 ± 2^a^	162 ± 2^c^	0.077 ± 0.004^d^	21.16 ± 0.38b^c^
D1	20/40	15	65	50.0 ± 1.5^d^	200 ± 4^b^	160 ± 3^d^	0.080 ± 0.003^c^	22.13 ± 0.40^c^
D2	20/40	25	65	48.0 ± 1.0^e^	195 ± 5^b^	155 ± 3^e^	0.085 ± 0.002^b^	22.57 ± 0.38^c^
D3	20/40	35	65	47.0 ± 1.0^e^	190 ± 5^b^	150 ± 3^e^	0.085 ± 0.002^b^	21.72 ± 0.37b^c^
T1	20/40/60	15	65	45.0 ± 1.2^f^	170 ± 3^c^	140 ± 2^f^	0.090 ± 0.003^ab^	20.87 ± 0.54^b^
T2	20/40/60	25	65	42.0 ± 1.3^g^	160 ± 6^c^	128 ± 4^g^	0.095 ± 0.003^a^	22.29 ± 0.48^c^
T3	20/40/60	35	65	40.8 ± 1.5^g^	153 ± 6^c^	120 ± 3^g^	0.098 ± 0.004^a^	21.74 ± 0.45b^c^

### 2.4 Starch isolation

The starch isolation method was slightly modified from the previously reported procedure by Bao, Ying, Zhou, Xu, Wu, Xu and Pang [14]. Frozen dough was thawed in a temperature and humidity-controlled chamber (RH 85 %, 35 °C) for 1 h. The thawed dough was then kneaded and hand-washed in a 2.57 % NaCl solution at a mass ratio of 1:3. The resulting starch slurry was passed through a 100-mesh filter cloth. After filtering, the starch slurry was centrifuged at 3000 × g for 10 min, and the upper pigmented layer was carefully removed. The starch was further purified by resuspension in distilled water and repeated centrifugation, then dried in a blast dryer at 40 °C for 48 h.

### 2.5 Swelling power and solubility

The swelling power and solubility of various samples were evaluated using the procedure described by [15]. A 1 % aqueous wheat suspension (100 ml) was heated to 95 °C in a water bath while continuously stirring for 1 h. The mixture was then cooled to 30 °C for 30 min and transferred to pre-weighed centrifuge tubes. The tubes were centrifuged at 3000 × g for 10 min, and the weight of the sediment was recorded to measure solubility. The supernatants were transferred to petri dishes and dried in a hot oven at 110 °C for 12 h. The dry solids were weighed after cooling to room temperature in a desiccator.

### 2.6 Particle size

The particle size distribution of wheat was determined using a laser-light particle size analyzer (S3500, Microtrac Inc., USA) equipped with a dry sample delivery system (Microtrac Turbotrac SDC, Microtrac Inc., USA) [16].

### 2.7 Amylose content

The amylose content was determined using the iodine binding method following the protocols described by [17]. Absorbance readings were measured at 620 nm. A standard curve was established using varying proportions of amylose standards. A regression equation was derived from the standard curve correlating amylose content with the difference in absorbance ABS620.

### 2.8 Morphological characteristics

Scanning electron micrographs were captured using a scanning electron microscope (Model EVOLS10 ZEISS, Oberkochen, Germany. To prepare the sample, wheat was suspended in ethanol to create a 1 % suspension. Next, a drop of this wheat-ethanol mixture was placed on an aluminium stub tape. Finally, the wheat was coated with a gold–palladium alloy [18].

### 2.9 Crystalline properties

#### 2.9.1 X-ray diffraction

X-ray diffractograms of wheat frozen dough samples were measured using Japan's Rigaku Miniflex analytical X-ray diffractometer. The device was equipped with a Cu-Kα source with a wavelength of λ = 1.54 Å. It operated at 45 kV and 40 mA. The X-ray diffractograms were obtained at a temperature of 25 °C within the 2θ angle range of 4–30 °C. A step size of 0.02 and a scan speed of 10 s were used. The data from MS Excel was exported to Origin Pro 8E software from Origin Lab in the USA to create graphs. The different wheat crystalline index (%) and d-spacing index were calculated using the methods described by [4].

#### 2.9.2 Fourier transform infrared spectroscopy

FTIR spectrum was obtained using a Fourier transform spectrophotometer, PerkinElmer FTIR-C92035, USA, with a wave number ranging from 400 to 4000 cm^−1^, employing the KBr pellet method [19].

#### 2.9.3 Differential scanning calorimeter

The thermal properties of all starch samples were analyzed using a DSC (DSC 214, Netzsch Instrument Co. Ltd, Germany), with slight modifications based on the method by Yang, Zheng, Li, Pan, Huang, Zhao and Ai [20]. A 3 mg starch sample was mixed with 6 μL of water, sealed in an aluminum DSC pan, and then stored at 4 °C for 24 h. An empty aluminum pan was used as a reference for each measurement [21]. The thermal analysis was performed by heating the samples from 30 °C to 90 °C at 10 °C per minute under a 40 mL/min nitrogen flow. Each experiment was conducted in triplicate for every sample.

### 2.10 Dynamic rheological properties

The rheometer equipped with parallel-plate geometry (PP-40) was used to determine. The changes in the viscoelastic properties of wheat frozen dough paste for retrogradation, following the method proposed by Liu et al. [22]. The gap, stress, and frequency were set at 1.0 mm, 1 Pa, and 1.0 rad/s to ensure measurements within the linear viscoelastic range. To prepare the wheat pastes, 20 % w/w wheat suspensions were stirred in sealed vials at room temperature for 1 h. Subsequently, the suspensions were cooked in a water bath set at 95 °C for 30 min, with vortex shaking for 10 s at 2-minute intervals. The cooked wheat pastes were promptly transferred between the plates of the preheated rheometer, which had been set to a temperature of 90 °C. The pastes were then cooled from 90 to 10 °C at a rate of 2.5 °C/min and held at 10 °C for 30 min. Throughout this process, the storage (G') and loss (G'') moduli of the cooked wheat pastes were recorded.

### 2.11 Pasting properties

The pasting properties of the samples were assessed using a Rapid Visco Analyzer (TecMaster, Perten Instruments, North Ryde BC, NSW, Australia). A 3 g sample was combined with 25 g of deionized water in an RVA canister. The analysis was conducted over 13 min. Initially, the slurry was stirred at 960 rpm for 10 s, then reduced to 160 rpm. The temperature was set to 50 °C for the first minute, followed by an increase to 95 °C at a rate of 6 °C/min. After maintaining 95 °C for 5 min, the temperature decreased to 50 °C at 6 °C/min and was held there for 2 min [23]. The parameters measured included peak viscosity (PV), trough viscosity (TV), breakdown, final viscosity (FV), and setback.

### 2.12 In vitro digestibility of wheat frozen dough

The in vitro digestibility of wheat was determined using a method based on [24], with minor modifications. The wheat was then divided into three categories based on the digestion rates: rapid digestible starch (RDS), slowly digestible starch (SDS), and resistant starch (RS).

### 2.13 Statistical analysis

The data presented in all tables are the averages of three independent observations. Results were analyzed using Analysis of Variance (ANOVA) and are expressed with the standard error of the mean. Averages were compared using Fisher's least significant difference (LSD) test, with a p-value of less than 0.05, which was considered statistically significant.

## 3 Results and discussion

### 3.1 Solubility and swelling power

**Fig. 1:**
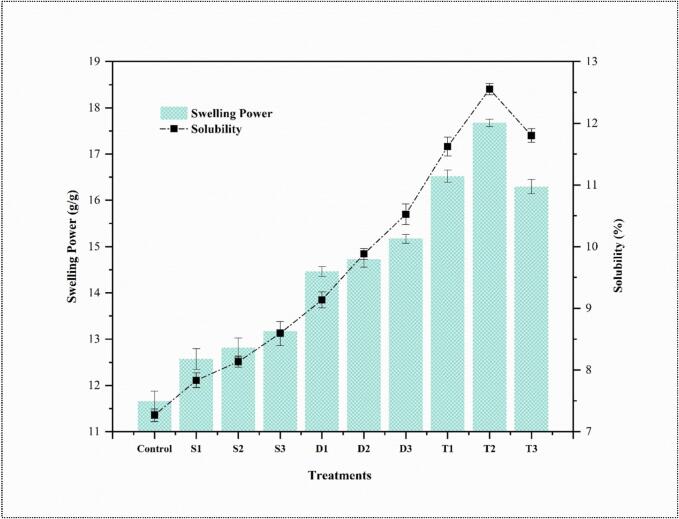
Swelling power and solubility percentage of starch samples subjected to different ultrasound treatments. The control sample exhibits the lowest values for both swelling power and solubility, while ultrasound treatments significantly enhance both parameters. Treatments T2 and T3 show the highest swelling power, followed by T1, indicating the impact of higher-intensity ultrasound. Similarly, solubility is maximized in T2, T3, and T1 treatments, suggesting greater granule disruption. Error bars represent standard deviations, and different letters above the bars indicate statistically significant differences between treatments (p < 0.05).

Fig. 1 shows the SP and solubility of both native and modified wheat frozen dough. US significantly (p < 0.05) increased the SP and solubility of wheat frozen dough by increasing the treatment time and frequencies. The highest SP and solubility values were observed in the T2-treated sample, which can be attributed to its higher phosphate group content on the amylopectin. Phosphate groups weaken the bonding within the crystalline domain, leading to increased granule hydration [25].

The US cavitation effectively disrupts the dough's protein and starch matrix in the T2-treated sample, ultimately increasing solubility. The optimal duration was achieved in the T2-treated sample, where the US maximizes solubility without causing excessive degradation, which might happen in prolonged exposure, such as in T3. The US explicitly disrupts the amorphous regions of wheat granules due to their lower structural integrity, where the amylose is located, compared to the crystalline state of amylopectin. It leads to the release of amylose into the aqueous medium, thereby increasing solubility. The US may also alter the physical geometry of pores and channels on the surface of wheat granules, allowing water molecules to penetrate more easily and increasing granule solubility [26]. US disintegrates intermolecular bonds, disrupts the crystalline molecular structure of wheat, causes water molecules to bind with the free hydroxyl groups of amylose and amylopectin through hydrogen bonds, breaking clusters of wheat granules, and induces structural changes, thereby increasing granule absorption [27]. The increased SP observed in the T2-treated sample suggests that the multifrequency US treatment at this specific duration optimizes the hydration capacity of the starch granules in the dough. The US may facilitate the breakdown of starch granules, making them more accessible to water, thereby increasing their SP.

The US induces cavitation, where microbubbles form and collapse rapidly, which can disrupt the molecular structure of the starch in the dough, promoting excellent water absorption. The US might also partially gelatinize the starch, enhancing its ability to swell when absorbed by water [7]. The optimal swelling observed in T2 could be due to the US exposure being enough to increase gelatinization without causing excessive damage to the starch structure. The US may also weaken the protein matrix surrounding the starch granules, allowing more water to penetrate and causing increased swelling [28]. The decreased SP in the T3-treated sample compared to T2 indicates that prolonged US exposure might lead to over-processing, where the starch granules or proteins are excessively broken down, reducing their ability to retain water. The T2 is an optimal treatment for increasing the SP and solubility, potentially improving the dough's functional textural and processing qualities of the wheat frozen dough.

### 3.2 Particle size distribution

Table 1 presents the PSD of wheat flour frozen dough starch subjected to different US treatments at multiple frequencies. The results show a significant decrease in PSD with increasing treatment time and frequency than the control sample. The highest reduction in D[3,4] D _[0.1]_ and D_[0.5]_ was observed in T3, which suggests that the multifrequency US modifies the particle structure, effectively potentially enhancing dough properties through better hydration and gluten development. This reduction in PSD is likely due to cavitation effects induced by the US, which disrupts the granule structure and results in finer starch particles. It also increases surface area and possibly alters functional properties such as solubility and digestibility [29]. The reduction in PSD with increasing US treatment time and frequency suggests that higher energy input breaks down starch granules more effectively. The decrease in D 0.1 and D0.5 values further emphasizes the efficacy of the US in promoting a more homogeneous PSD. These findings align with Raza, Zhou, Cheng, He and Wang [30], who noted that smaller and more uniformly sized particles improved the rheological properties of dough, ultimately enhancing its baking performance.

Results indicate that the US treatments reduce the average PSD and contribute to a more uniform distribution, as reflected by the decreasing Span Index SI values. Treatments T1, T2, and T3 show higher SI values, suggesting a broader distribution in smaller particle sizes. It could be attributed to the breakdown of larger particles and the formation of smaller aggregates during US processing. US treatments generally lead to a reduction in particle size, which can enhance the functional properties of flours [31]. In buckwheat flour, US treatment at different solid-to-liquid ratios resulted in varied particle sizes, with higher dilution leading to smaller particle size fractions [32]. The method of water removal post-US treatment significantly affects the final particle size and properties of the flour [33]. This finding aligns with Pan, Zhang, Mintah, Xu, Dabbour, Cheng, Dai, He and Ma [34], who observed that the US increased the SI and altered the structure of food particles, enhancing their functional properties such as water absorption and texture. Moreover, the implications of these PDS changes are significant for the processing and final quality of baked products Wei, Li and Zhu [7]. As suggested, finer particles can enhance the hydration capacity of flour, leading to better dough extensibility and improved texture in baked goods.

### 3.3 Amylose content

In Table 1, the analysis of amylose content across various ultrasound treatments reveals significant variations influenced by frequency and duration. The control sample exhibited an amylose content of (19.31 ± 0.54 %), reflecting the native properties of wheat starch without ultrasound treatment. Single-frequency ultrasound (S1, S2, S3) showed a notable increase in amylose content with S2, achieving the highest value (22.09 ± 0.43 %), indicating that moderate treatment enhances the release of amylose due to cavitation effects. Prolonged exposure in S3 resulted in a slight decrease (21.16 ± 0.38 %) compared to S2, suggesting a potential equilibrium or minor reformation of amylopectin from shorter amylose chains. Karwarsa et al. Investigated the effects of ultrasonication on wheat starches and found an increase in the amylose content [35]. Dual-frequency ultrasound (D1, D2, D3) demonstrated consistent improvement in amylose content, with D2 reaching the highest value (22.57 ± 0.38 %) among all treatments. This suggests that dual-frequency ultrasound optimally disrupts amylopectin while enhancing amylose solubility through enhanced cavitation synergy. However, prolonged exposure in D3 (21.72 ± 0.37 %) led to a slight decrease, possibly due to structural reorganization or degradation effects. Triple-frequency ultrasound (T1, T2, T3) also improved amylose content, with T2 achieving the second highest value (22.29 ± 0.48 %). This indicates that triple-frequency ultrasound can effectively balance amylose release and amylopectin breakdown. T3 (21.74 ± 0.45 %) showed a similar trend of slight reduction with extended treatment. A study on corn starch subjected to ultrasound treatment observed an increase in leached amylose, which plays a crucial role in the gelation and retrogradation of starch pastes [36]. These results suggest that the optimized ultrasound conditions S2, D2, and T2 maximize amylose release, enhancing the functional properties of wheat starch. The findings highlight the potential of ultrasound technology, particularly dual and triple frequencies, to enhance starch modification, aligning with the study's goal of improving rheological and functional properties of frozen wheat dough starch.

### 3.4 Morphological characteristics

**Fig. 2:**
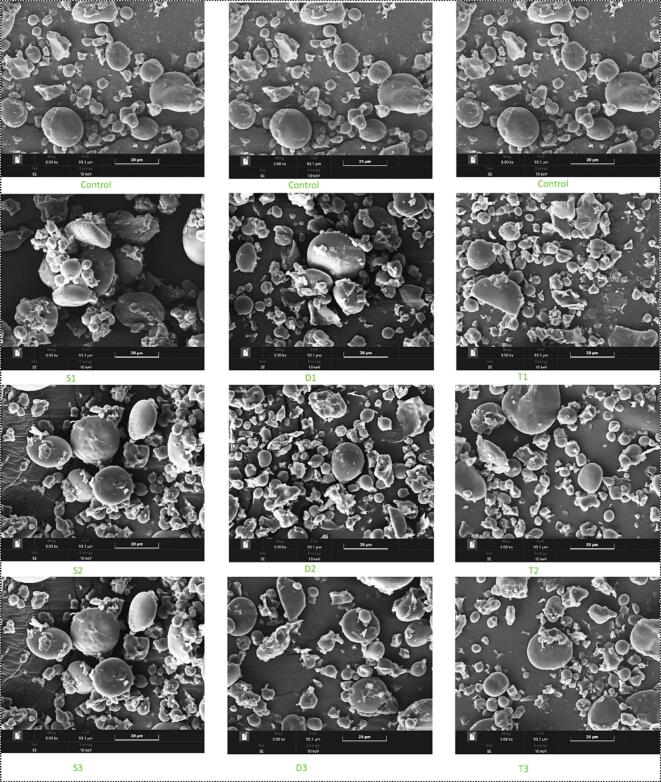
Scanning Electron Microscopy SEM images of wheat starch granules from control and ultrasound-treated samples. The images show structural changes at a magnification of 3.00 kx. The treatments include single frequency (S1, S2, S3), dual frequency (D1, D2, D3) and triple frequency (T1, T2, T3) ultrasound Control samples top row exhibit intact and smooth starch granules Ultrasound-treated samples middle and bottom rows show varying degrees of granule disruption and surface roughness indicating the impact of ultrasound treatments on starch morphology The treatment intensities affect granule size shape and surface structure as seen from the increased fragmentation and surface deformation in treated samples This description summarizes the visual differences observed across treatments.

The morphological changes in wheat frozen dough starch resulting from multifrequency US treatment are vividly demonstrated in Fig. 2. Among the various treatments, T2 showed significant improvements in the structural integrity and functionality of the wheat granules, making it the optimal treatment. US induces morphological changes in wheat granules primarily through the collapse of cavitation bubbles, which generate high-pressure gradients and intense shear forces, causing rupture and mechanical damage to the starch granules. These forces can break polymer chains within the granules, resulting in a more porous and less dense structure. The surface of untreated wheat granules typically appears smooth and compact, but post-US, depressions, pores, and channels are formed, significantly altering the granules' morphology [37].

In the T2 treatment, the granules exhibited prominent depressions and channels compared to other treatments, indicating more extensive structural modification. It is likely due to the optimized combination of US frequency and treatment time, which enhances the cavitation effect and leads to more pronounced morphological changes. As Ahmed, Xu, Farooq, Manzoor, Awad, Ashraf, Tufail and Abdi [38] reported, the granules showed small fissures and depressions on the surface of maize granules under optimal conditions. Through multifrequency US, T2 is the most effective treatment for enhancing wheat frozen dough starch's morphological and functional properties. The mechanism driving these changes is rooted in the intense shear forces and cavitation effects that the US imparts on the granules, leading to significant structural modifications. This treatment improves the granule structure and enhances the dough's overall functionality, making it a promising approach for producing high-quality frozen dough products. Future research should optimize US parameters to maximize these benefits while minimizing potential degradation effects.

### 3.5 Crystalline

#### 3.5.1 X-ray diffraction

**Fig. 3:**
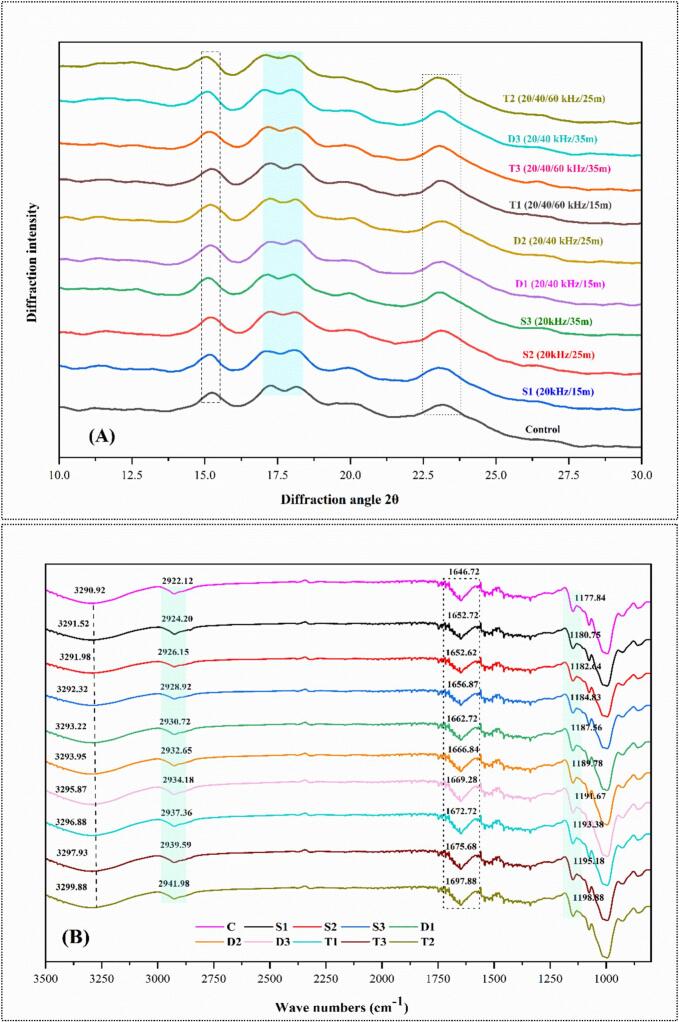
**(A)** The individual presentation of XRD, and **(B)** individual peaks representation of single dual and triple frequency and time.

XRD analysis was performed to assess the impact of multifrequency US treatment on the crystalline structure of wheat frozen dough starch, as shown in Fig. 3A. All samples exhibited the characteristic A-type crystalline structure with prominent peaks at 15.2°, 17.8° (doublet peak), 18.5°, and 22.7°, indicative of typical cereal starches. The d-spacing values obtained were around 5.86, 4.91, 4.45, and 3.87 Å, corresponding to an A-type crystalline arrangement consistent with literature reports for wheat starch. The crystalline index across the treatments ranged from 32.08 to 44.39 %, demonstrating some variability, although the changes were minimal. The T2 treatment at 20/40/60 kHz for 25 min was identified as the optimal condition, leading to a higher crystalline index than other treatments.

Despite the application of US, the XRD patterns overall showed no significant alteration in the crystallinity or polymorphic type, suggesting that US treatment primarily affects the amorphous regions of the starch granules while leaving the crystalline structure relatively intact. The Relative Crystallinity Index (RCI) was estimated from XRD patterns, showing a clear decline from 30.5 % (Control) to 20.3 % (T3), indicating a significant reduction in long-range order due to increased sonication frequency and time (Table S1). Sonication can lead to a decrease in the degree of crystallinity, as observed in chitosan, where prolonged sonication time and higher temperatures resulted in reduced crystallinity without significant changes in chemical structure [39]. The application of multifrequency US, particularly at the optimal T2 condition, likely enhances the reorganization of starch molecules within the amorphous regions, leading to improved packing and slight increases in crystallinity. US waves generate cavitation effects, which can cause micro-movements within the starch granules [40]. These movements facilitate the alignment of polymer chains in the amorphous regions, potentially contributing to the observed increase in crystallinity under the T2 treatment. The minimal impact on the overall crystalline structure observed in this study aligns with previous findings, where the US had negligible effects on the polymorph type of wheat starches [41]. The varying susceptibility to US among granules may be attributed to differences in the packing of crystalline and amorphous components, leading to the observed variability in diffraction intensities [42]. In conclusion, while multifrequency US at the T2 condition enhances the amorphous packing of starch granules, it does not significantly alter the overall crystalline structure, indicating its potential as a non-invasive method for improving the functional properties of wheat frozen dough without compromising its structural integrity.

#### 3.5.2 Fourier transform infrared spectroscopy

The FTIR spectra revealed characteristic peaks associated with the molecular vibrations within the starch granules (Fig. 3B). Fig. 3B shows that the key peaks were observed in the regions corresponding to O-H and C-H bond stretching and the vibrations of the anhydrous glucose ring. The broad peaks observed between 2922 to 3290 cm^−1^ correspond to O-H bond stretching in the hydroxyl groups. The short-range order, represented by the A_1047_/A_1022_ FTIR ratio, decreased from 1.04 (Control) to 0.89 (T3), demonstrating disruption in the double-helical structure (Table S2). The decrease in the A_1047_/A_1022_ ratio can be linked to chemical modifications such as acetification, which reduces crystallinity by altering the starch's molecular structure, as observed in modified starch studies [43]. After US treatments, the increase in peak intensity indicates that US waves enhance the microstructure of wheat, leading to better retention of bound water. This retention is likely due to the disruption of the granular structure, which exposes more hydroxyl groups and facilitates water interaction [44].

Peaks in the 1000 to 1150 cm^−1^ range were assigned to C-H bond stretching within the glucose units. Ultrasound treatment intensified these peaks, indicating that the application of ultrasound may disrupt the crystalline regions, increasing the accessibility of these functional groups to infrared light [25]. The peaks observed at 1550 to 1650 cm-^1^ were associated with water in the amorphous regions of the starch. The enhanced FTIR absorbance ratios observed in the T2 treatment suggest that multifrequency US (20/40/60) kHz treatment at 25 min effectively disrupts the amorphous regions of the starch granules, leading to increased water retention and improved molecular alignment. The variation in peak intensity across different treatments indicates changes in the amorphous content, which correlates with the US treatment disrupting the granular structure and increasing amorphous content [45]. The cavitation effects generated by the US create localized high-pressure zones, which can disrupt the weak hydrogen bonds in the amorphous regions, thereby increasing the interaction of water molecules with the starch matrix [46]. Results indicate that the T2 treatment was an optimal condition for improving the functional properties of wheat frozen dough, particularly in enhancing water retention and short-range molecular order while maintaining overall starch integrity [41].

#### 3.5.3 Diffraction scanning calorimetry

The DSC analysis of wheat frozen dough starch treated with multifrequency US is presented in Table 2. The parameters evaluated include onset temperature, peak temperatures, enthalpy change (ΔH), conclusion temperature, heat flow, and crystallinity. The results indicate that the T2 and T3 treatments exhibited the highest onset temperatures of 54.5 °C and 54.0 °C, respectively, suggesting that these samples begin melting at slightly higher temperatures than the control sample (52.5 °C). These results indicate that the dough's crystalline structure in the US-treated samples, particularly T2, is more stable and possibly more resistant to initial thermal disruption. Such stability can be attributed to more uniform crystallite distribution or improved protein-starch interactions induced by US treatment.

**Table 2:** Impact of multifrequency ultrasound on the diffraction scanning calorimetry.

Treatment	Onset Temp (°C)	Peak Temp 1 (°C)	Peak Temp 2 (°C)	ΔH (J/g)	Conclusion Temp (°C)	Heat Flow (W/g)	Crystallinity (%)
Control	53.5 ± 0.4^ab^	57.7 ± 0.5^a^	63.3 ± 0.3^a^	6.57 ± 0.12^a^	44.17 ± 0.9^a^	0.88 ± 0.01^a^	13.3 ± 0.4^d^
S1	53.2 ± 0.1^b^	58.0 ± 0.5^a^	63.3 ± 0.0^a^	6.27 ± 0.03^b^	44.15 ± 0.2^a^	0.81 ± 0.01^a^	13.2 ± 0.2^d^
S2	53.8 ± 0.5^ab^	58.0 ± 0.2^a^	63.3 ± 0.3^a^	5.87 ± 0.05^c^	44.10 ± 0.2^a^	0.82 ± 0.06^a^	14.4 ± 0.9^cd^
S3	54.0 ± 0.1^ab^	58.4 ± 0.3^a^	63.3 ± 0.1^a^	5.67 ± 0.10^d^	43.19 ± 1.0^a^	0.88 ± 0.07^a^	15.7 ± 1.0^c^
D1	53.9 ± 0.8^ab^	57.7 ± 0.6^a^	63.3 ± 0.7^a^	4.96 ± 0.03^e^	44.10 ± 0.2^a^	0.86 ± 0.02^a^	17.7 ± 0.3^b^
D2	54.8 ± 0.5^a^	57.8 ± 0.4^a^	62.7 ± 0.1^b^	4.22 ± 0.02^f^	43.19 ± 0.4^a^	0.85 ± 0.01a	20.3 ± 0.1^a^
D3	54.0 ± 0.7^a^	58.5 ± 0.3^a^	62.8 ± 0.2^b^	4.10 ± 0.04^f^	43.17 ± 0.5^a^	0.85 ± 0.02^a^	21.0 ± 0.3^a^
T1	54.2 ± 0.5^a^	58.3 ± 0.5^a^	62.6 ± 0.3^b^	4.05 ± 0.02^f^	43.16 ± 0.4^a^	0.84 ± 0.03^a^	21.5 ± 0.2^a^
T2	54.5 ± 0.6^a^	58.0 ± 0.4^a^	62.5 ± 0.4^b^	4.00 ± 0.03^f^	43.15 ± 0.5^a^	0.83 ± 0.02^a^	22.0 ± 0.4^a^
T3	54.0 ± 0.8^a^	57.8 ± 0.3^a^	62.3 ± 0.5^b^	3.95 ± 0.05^g^	43.14 ± 0.6^a^	0.82 ± 0.04^a^	21.8 ± 0.5^a^

**Notes: Onset Temp (°C):** The temperature at which the phase change (e.g., crystallization) begins, **Peak Temp 1 & 2 (°C):** Temperatures at which maximum heat absorption or release occurs, indicating phase transitions, **ΔH (J/g):** The enthalpy change associated with the transition, indicating energy required for the phase change. **Conclusion Temp (°C):** The temperature at which the phase change ends, and **Heat Flow (W/g):** The rate of energy flow during the transition, which affects texture and stability.

Two peak temperatures were noted in the DSC analysis, indicating the presence of multiple crystalline phases within the dough. T2 again shows lower peak temperatures (58.0 °C and 62.5 °C) compared to the control sample (58.7 °C and 64.3 °C). A lower peak temperature could signify that less energy is required to melt the crystalline structures, possibly due to US treatment breaking down larger crystallites into smaller, more uniform ones. The enthalpy change directly measures the energy required for the phase transition. T2 displayed the lowest ΔH (4.00 J/g), significantly lower than the control (6.59 J/g). The decrease in enthalpy changes shows that the US treatment effectively alters the dough's microstructure, making it more efficient in transitioning between phases. The lower enthalpy indicates that less energy is needed for the dough to undergo melting, potentially due to increased molecular mobility within the dough matrix, which is often enhanced by the US through cavitation phenomena [7].

Results indicate that the T2 sample had the lowest conclusion temperature, 43.5 °C, compared to the control sample (44.7 °C). This drop indicates a more uniform melting process where the dough structure is less heterogeneous, leading to a quicker transition from solid to molten phase. This uniformity is likely a result of the US's ability to disrupt and redistribute the dough components, leading to a more consistent internal structure. The heat flow rate results describe the dough's energy dynamics; in the present study, all the samples exhibited lower heat flow values than the control sample, indicating that the energy distribution during phase transitions is more efficient. This efficiency could result from the reduced viscosity and improved homogeneity of the dough, as US treatment is known to enhance the dispersion of particles and other constituents within a matrix [47].

Crystallinity influences the texture and quality of frozen dough, and our results showed that the T2 sample had the highest crystallinity, 22.0 %, then the control sample, 13.4 %. The increase in crystallinity associated with US treatment enhances the ordered structure within the dough, likely contributing to better texture and improved mechanical properties upon baking. Also, the increase in crystallinity might be more likely due to the US, which induced the alignment of starch molecules and possibly improved protein crosslinking, as noted by Yang, Kong, Zheng, Sun, Chen, Liu, Zhang, Fang, Tian and Ye [48]. Conclusively, the DSC results demonstrate that multifrequency US treatments, particularly T2, significantly improve the thermal properties of wheat frozen dough. All these changes can be attributed to the physical effects of the US, particularly the cavitation effect, which generates microbubbles that implode, causing intense localized pressure and temperature changes to disrupt large starch granules and protein networks, resulting in a finer, more homogeneous dough structure [42]. These changes can contribute to improved dough quality, better texture, and potentially longer shelf life, making US treatment a promising technique for frozen dough processing.

### 3.6 Rheological properties

**Fig. 4:**
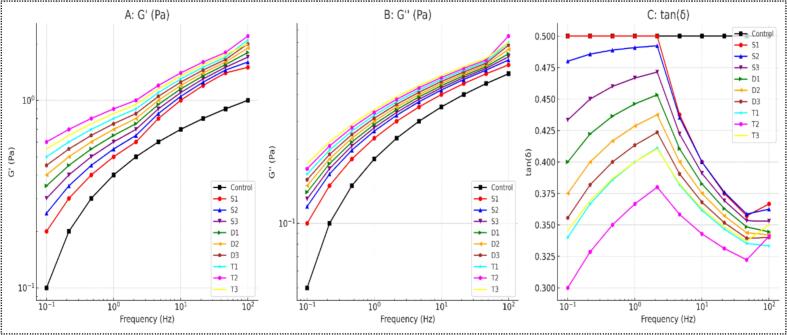
Rheological properties of wheat frozen dough starch samples subjected to different ultrasound treatments **(A)** Storage modulus (G'), **(B)** Loss modulus (G''), and **(C)** Damping factor (tan δ) are plotted as functions of frequency for control and ultrasound treated dough samples. The ultrasound treatments include single frequency (S1, S2, S3), dual frequency (D1, D2, D3), and triple frequency (T1, T2, T3). These curves demonstrate the frequency-dependent viscoelastic behavior of the dough, with all ultrasound-treated samples showing improved rheological properties compared to the control.

The rheological properties (storage modulus (G'), loss modulus (G''), and loss tangent (tan δ)) of wheat frozen dough starch were measured to assess the viscoelastic properties of the starch samples, as shown in Fig. 4 A, B, and C, respectively. The G' represents the elastic behavior of the dough and its ability to store energy when deformed. Fig. 4A shows that G' increased with US frequency across all treatments, indicating an enhanced elastic network structure within the starch granules. Among all the treatments, T2 showed the highest G' values and significantly strengthened the starch matrix, likely due to the optimized balance between US treatment time and power. He, Chen, Liu, Teng and Li [49] reported that moderate US treatment can disrupt starch granule surfaces and enhance water absorption, leading to a more extensive gelatinization process and increased G' values. The US-induced cavitation might promote the formation of a denser gel network by increasing the interaction between starch molecules. The T2 treatment's superior performance may be attributed to an optimal combination of cavitation effects that reinforce the structural integrity of the starch network without causing excessive degradation [38].

The loss modulus (G''), which indicates the viscous behavior or energy dissipated as heat, also increased with US frequency across all treatments (Fig. 4B). The magnitude of G'' was lower than G' across all treatments, reflecting a predominantly elastic dough behavior. T2 again exhibited the highest G'' values, indicating that the treatment enhanced elasticity but also slightly increased the viscous properties of the dough. The increase in G'' in the T2-treated sample might be due to US-induced partial disruption of the starch granules and amylose lipid complexes, which could lead to the release of amylose chains into the matrix. This release contributes to the entanglement and interaction between amylose molecules, enhancing the dough's viscous and elastic properties [50].

The loss tangent (tan δ = G''/G') provides a viscous-to-elastic behavior ratio, giving insight into the overall viscoelastic nature of the starch. In Fig. 4C, tan δ values show a decreasing trend with increasing US frequency, indicating a more solid-like behavior at higher frequencies. The T2 treatment resulted in the lowest tan δ values, showing the most solid-like behavior among all treatments. This lower tan δ indicates a highly structured and interconnected starch network, less prone to deformation under stress. Such behavior can be attributed to the cavitation effects of US, which can modify the molecular structure of starch without disrupting its integrity excessively. [38] reported that US treatment at specific frequencies and treatment times can enhance the viscoelastic properties of starch by creating a more homogeneous and resilient starch matrix. Conclusively, the US cavitation phenomena led to micro-jet impacts and localized shear forces that modified the starch granules and their interactions. The resultant changes in starch morphology and molecular interactions could explain the increased gel strength (higher G') and reduced loss tangent (lower tan δ), suggesting a more robust gel network.

### 3.7 Pasting properties

Multifrequency US treatments significantly affect the pasting properties of wheat dough starch, as shown in Table 3. Results indicate that the peak, trough, and final viscosities significantly increased (p < 0.05) among all treatments by increasing frequency and treatment time, while breakdown and setback viscosities had a non-significant impact. The US treatment facilitated water absorption and starch gelatinization, resulting in higher peak viscosities, which are beneficial in processes requiring high viscosity, such as dough formulations for frozen products. The retrogradation behavior of starch is a critical factor in determining the quality and shelf life of starch-based food products [51]. Lower final viscosities and setbacks in starch samples treated at higher frequencies and longer times indicate reduced retrogradation potential, which is beneficial for maintaining textural quality in frozen products.

**Table 3:** Pasting properties of wheat frozen dough starch treated and without treated samples.

Treatment	Peak Viscosity (cP)	Trough Viscosity (cP)	Breakdown (cP)	Final Viscosity (cP)	Setback (cP)	Pasting Temperature (°C)
Control	310 ± 15^c^	200 ± 10^c^	110 ± 5^c^	250 ± 12^c^	50 ± 4^c^	72.5 ± 0.5^c^
S1	320 ± 18^bc^	210 ± 11^bc^	111 ± 6^c^	260 ± 14^bc^	51 ± 5^c^	73.0 ± 0.6^c^
S2	330 ± 20 ^bc^	220 ± 13^b^	111 ± 7^c^	270 ± 15^bc^	51 ± 4^c^	73.5 ± 0.4^bc^
3	340 ± 19^b^	230 ± 12^b^	112 ± 8^c^	280 ± 16^b^	53 ± 3^c^	74.0 ± 0.5^b^
D1	350 ± 21_b_	240 ± 14^b^	113 ± 6^c^	290 ± 17^b^	54 ± 4^c^	74.5 ± 0.5^b^
D2	360 ± 23 ^ab^	250 ± 15^ab^	113 ± 7^c^	300 ± 18^ab^	54 ± 5^c^	75.0 ± 0.4^ab^
D3	370 ± 24^ab^	260 ± 16^ab^	114 ± 8^c^	310 ± 19^ab^	55 ± 6^c^	75.5 ± 0.6^ab^
T1	380 ± 25^a^	270 ± 17^a^	115 ± 6^c^	320 ± 20^a^	56 ± 5^c^	76.0 ± 0.5^a^
T2	390 ± 26^a^	280 ± 18^a^	116 ± 7^c^	330 ± 21 ^a^	57 ± 4^c^	76.5 ± 0.4^a^
T3	385 ± 25^a^	275 ± 16 ^a^	115 ± 6^c^	325 ± 19 ^a^	55 ± 5^c^	76.2 ± 0.5 ^a^

Higher pasting temperatures in more intensely treated samples suggest that more energy is required for gelatinization, possibly due to changes in the crystalline structure or increased molecular interactions post-treatment. Retrogradation and viscosity: Lower final viscosities and setbacks are associated with reduced retrogradation, as seen in low-viscosity potato starch (LVPS) compared to native potato starch (NPS) [52]. The addition of tara gum and k-carrageen can decrease setback viscosity and pasting temperature, indicating retrogradation. In addition, starches decrease setback and breakdown values, further inhibiting retrogradation [53]. Pasting temperature and gelatinization: Higher pasting temperature in treated starches suggests increased energy requirements for gelatinization, possibly due to partial degradation of crystalline regions or enhanced molecular interactions. Sugar like sucrose can evaluate pasting temperatures, while glucose and fructose mixtures affect starch properties differently, influencing retrogradation [54].

Factors influencing retrogradation: Storage conditions, such as time and temperature, significantly affect retrogradation, with lower temperatures and longer durations increasing the degree of retrogradation in rice starch. Protein-starch interactions can either inhibit or promote retrogradation, depending on the nature of the protein residues involved [55]. While the reduction in retrogradation is generally advantageous for maintaining the quality of frozen products, it is important to consider the specific application and desired textural properties. For instance, in some cases, a certain degree of retrogradation might be beneficial for achieving the desired firmness or texture in backed goods or pasta. Therefore, understanding the balance between retrogradation and product quality is essential for optimizing food formulations.

### 3.8 In vitro digestibility

**Fig. 5:**
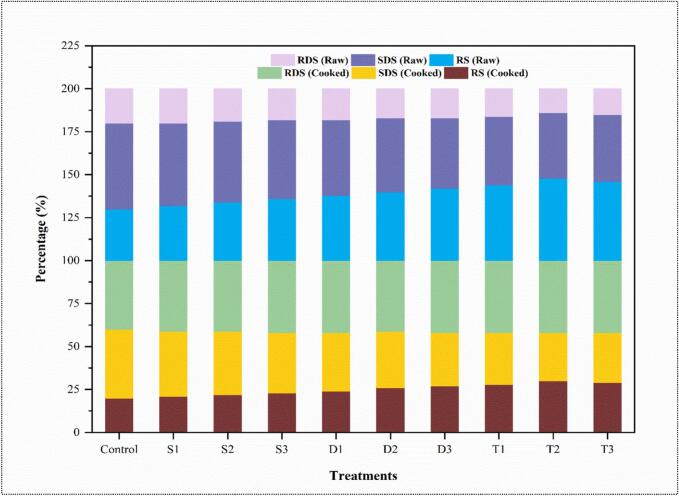
The distribution of rapidly digestible starch (RDS), slowly digestible starch (SDS), and resistant starch (RS) percentages in raw and cooked samples of wheat frozen dough subjected to different ultrasound treatments. The treatments include single frequency S1, S2, S3, dual frequency D1, D2, D3, and triple frequency T1, T2, T3, ultrasound applications. The control sample is also shown for comparison.

Fig. 5, the stacked column charts showing the digestibility of starch across different treatments in both raw and cooked forms, indicating significant variations in rapidly digestible starch (RDS), slowly digestible starch (SDS), and resistant starch (RS) contents. In raw form, the RDS content shows an increasing trend from Control (20 %) to T2 (30 %). The Control group has the lowest RDS, indicating that the untreated sample is less prone to rapid digestion. The highest RDS in T2 suggests that this treatment significantly enhances the proportion of starch rapidly available for digestion. It is likely due to structural changes in the starch granules, increasing their susceptibility to enzymatic hydrolysis. After cooking, the RDS content increases across all treatments, typically due to gelatinization. The increase is more pronounced in treatments with initially lower RDS in the raw state, particularly in T1 and T2, suggesting that these treatments enhance gelatinization, making the starch more digestible.

In raw form, the SDS content is highest in the control (40 %) and gradually decreases with the US treatment, with the lowest SDS contents observed in the T2 treatment (28 %). This inverse relationship between RDS and SDS suggests a conversion of slowly digestible starches into rapidly digestible forms through the treatments [56]. This shift indicates changes in the crystalline structure of starch, making it less resistant to enzymatic attack [57]. After cooking, the SDS content decreases across all the treatments but remains substantial in the Control and less-treated samples like S1 and S2. The cooked SDS levels in T1 and T2 are notably lower, reflecting their higher conversion to RDS. The RS content remains relatively stable across treatments in the raw state, hovering around 41–42 %, with minor fluctuations. Post-cooking, the RS content significantly (p < 0.05) decreased in T1, T2, and T3 treatments; the resistant starch often converts to more digestible forms upon cooking.

This could be advantageous for products targeting quick energy release but may not be desirable in contexts where sustained glucose release is needed, such as diabetic-friendly foods [58]. Moreover, the pronounced decrease in RS content upon cooking, especially in T2 and T3, raises considerations for the nutritional quality of these starches post-cooking. The loss of RS, which is beneficial for gut health due to its fermentability by colonic bacteria, could reduce the health benefits of these treatments. The treatments, especially T2, enhance the rapid digestibility of starch; they do so at the expense of slowly digestible and resistant starch fractions, which could influence both the metabolic and health outcomes of consuming such starches [59]. These findings highlight the need to balance starch modifications to achieve desired nutritional outcomes while maintaining overall health benefits.

## 4 Conclusion

This study confirms that multifrequency US is a groundbreaking physical method that significantly improves the wheat frozen dough functional, rheological, and ultra-structural properties. The results revealed that the US processing of wheat dough starch substantially improves product quality and processing efficiency. Specifically, the T2 treatment emerged as the most effective, optimizing the balance between US frequency and exposure time, resulting in notable morphological changes in the starch granules. The cavitation effects induced by US generate high-pressure gradients and shear forces, leading to mechanical disruption of the granules. This disruption enhanced the granules' porosity and surface morphology, improving the dough's functional properties, including swelling power and solubility. These changes were achieved without compromising the structural integrity of the granules, making the treatment both effective and reliable. Moreover, the US treatments increased the content of rapidly digestible starch RDS and resistant starch RS. The US-induced formation of short-chained amylose molecules likely contributed to these nutritional enhancements, making the treated wheat starch more suitable for various food products. In conclusion, applying the multifrequency US in wheat frozen dough processing offers the food industry a versatile tool to enhance product quality, reduce processing time, and achieve energy savings, all while supporting eco-friendly practices. The findings of this study highlight the potential for further optimization and application of ultrasound technology in wheat-based products, paving the way for future innovations in food processing.

## CRediT authorship contribution statement

**Zahoor Ahmed:** Writing – review & editing, Writing – original draft, Visualization, Methodology, Investigation, Conceptualization. **Abid Hussain:** Writing – review & editing, Visualization. **Umar Farooq:** Visualization, Investigation, Data curation. **Yibin Zhou:** Writing – review & editing, Investigation. **Isam A. Mohamed Ahmed:** Writing – review & editing. **Muhammad Waseem:** Software, Investigation. **Bin Xu:** Writing – review & editing, Writing – original draft, Supervision, Resources, Project administration, Funding acquisition. **Muhammad Faisal Manzoor:** Writing – review & editing, Writing – original draft, Visualization. **Robert Mugabi:** Writing – review & editing.

## Declaration of competing interest

The authors declare that they have no known competing financial interests or personal relationships that could have appeared to influence the work reported in this paper.

## References

[1] Zhang Q., Jin M., An D., Ahmed Z., Qi Y., Xu B. (2023). Modelling dried noodle quality: Contribution of starch and protein physicochemical properties of 32 wheat cultivars. Food Res. Int. (Ottawa, Ont.).

[2] Xu C., Li C., Li E., Gilbert R.G. (2024). Insights into wheat-starch biosynthesis from two-dimensional macromolecular structure. Carbohydr. Polym..

[3] Zhao Y., Wang J., He R., Ren Y., Fu J., Zeng Y., Zhang K., Zhong G. (2024). Integrative experimental and computational analysis of the impact of KGM's polymerization degree on wheat starch's pasting and retrogradation characteristics. Carbohydr. Polym..

[4] Li Y., Zhao F., Li C., Ban X., Gu Z., Li Z. (2022). Fine structures of added maltodextrin impact stability of frozen bread dough system. Carbohydr. Polym..

[5] Yang Z., Yu W., Xu D., Guo L., Wu F., Xu X. (2019). Impact of frozen storage on whole wheat starch and its A-Type and B-Type granules isolated from frozen dough. Carbohydr. Polym..

[6] Zhou Y., Petrova S.P., Edgar K.J. (2021). Chemical synthesis of polysaccharide-protein and polysaccharide-peptide conjugates: a review. Carbohydr. Polym..

[7] Wei Y., Li G., Zhu F. (2023). Impact of long-term ultrasound treatment on structural and physicochemical properties of starches differing in granule size. Carbohydr. Polym..

[8] Dong Y., Novo D.C., Mosquera-Giraldo L.I., Taylor L.S., Edgar K.J. (2019). Conjugation of bile esters to cellulose by olefin cross-metathesis: a strategy for accessing complex polysaccharide structures. Carbohydr. Polym..

[9] Ahmed Z., Uddin N., Latif A., Tufail T., Qayum A., Manzoor M.F., Khan K.A., Ashraf J., Khalid N., Xu B. (2024). Improving the quality and digestibility of wheat flour starch and protein for noodles through ultrasound, high hydrostatic pressure, and plasma technologies: a review. Int. J. Biol. Macromol..

[10] Ahmed Z., Faisal Manzoor M., Hussain A., Hanif M., Zia-Ud-Din, Zeng X.A. (2021). Study the impact of ultra-sonication and pulsed electric field on the quality of wheat plantlet juice through FTIR and SERS. Ultrason. Sonochem..

[11] Luo W., Sun D., Zhu Z., Wang Q. (2018). Improving freeze tolerance of yeast and dough properties for enhancing frozen dough quality - a review of effective methods. Trends Food Sci. Technol..

[12] Khadhraoui B., Ummat V., Tiwari B.K., Fabiano-Tixier A.S., Chemat F. (2021). Review of ultrasound combinations with hybrid and innovative techniques for extraction and processing of food and natural products. Ultrason. Sonochem..

[13] Yang Y.L., Guan E.Q., Zhang L.L., Pang J.Y., Li M.M., Bian K. (2021). Effects of vacuum degree, mixing speed, and water amount on the moisture distribution and rheological properties of wheat flour dough. J. Food Sci..

[14] Bao J., Ying Y., Zhou X., Xu Y., Wu P., Xu F., Pang Y. (2020). Relationships among starch biosynthesizing protein content, fine structure and functionality in rice. Carbohydr. Polym..

[15] Chen F., Sawada D., Hummel M., Sixta H., Budtova T. (2020). Swelling and dissolution kinetics of natural and man-made cellulose fibers in solvent power tuned ionic liquid. Cellul..

[16] Zhou T., Zhang L., Zhao R., Liu Q., Liu W., Hu H. (2022). Effects of particle size distribution of potato starch granules on rheological properties of model dough underwent multiple freezing-thawing cycles. Food Res. Int. (ottawa, Ont.).

[17] Vilaplana F., Hasjim J., Gilbert R.G. (2012). Amylose content in starches: toward optimal definition and validating experimental methods. Carbohydr. Polym..

[18] Chi C., Xu K., Wang H., Zhao L., Zhang Y., Chen B., Wang M. (2023). Deciphering multi-scale structures and pasting properties of wheat starch in frozen dough following different freezing rates. Food Chem..

[19] Ahmed Z., Manzoor M.F., Begum N., Khan A., Shah I., Farooq U., Siddique R., Zeng X., Rahaman A., Siddeeg A. (2019). Thermo-ultrasound-based sterilization approach for the quality improvement of wheat plantlets juice. Processes.

[20] Yang Y., Zheng S., Li Z., Pan Z., Huang Z., Zhao J., Ai Z. (2021). Influence of three types of freezing methods on physicochemical properties and digestibility of starch in frozen unfermented dough. Food Hydrocoll..

[21] Zhao A., Shi P., Yang R., Gu Z., Jiang D., Wang P. (2022). Isolation of novel wheat bran antifreeze polysaccharides and the cryoprotective effect on frozen dough quality. Food Hydrocoll..

[22] Liu Y., Leng Y., Xiao S., Zhang Y., Ding W., Ding B., Wu Y., Wang X., Fu Y. (2022). Effect of inulin with different degrees of polymerization on dough rheology, gelatinization, texture and protein composition properties of extruded flour products. LWT.

[23] Wang H., Xu K., Liu X., Zhang Y., Xie X., Zhang H. (2021). Understanding the structural, pasting and digestion properties of starch isolated from frozen wheat dough. Food Hydrocoll..

[24] Qi K., Yi X., Li C. (2022). Effects of endogenous macronutrients and processing conditions on starch digestibility in wheat bread. Carbohydr. Polym..

[25] Zhang J., Luo D., Xiang J., Xu W., Xu B., Li P., Huang J. (2021). Structural variations of wheat proteins under ultrasound treatment. J. Cereal Sci..

[26] Wu Z., Qiao D., Zhao S., Lin Q., Zhang B., Xie F. (2022). Nonthermal physical modification of starch: an overview of recent research into structure and property alterations. Int. J. Biol. Macromol..

[27] Hao Z., Xu H., Yu Y., Han S., Gu Z., Wang Y., Li C., Zhang Q., Deng C., Xiao Y., Liu Y., Liu K., Zheng M., Zhou Y., Yu Z. (2023). Preparation of the starch-lipid complexes by ultrasound treatment: exploring the interactions using molecular docking. Int. J. Biol. Macromol..

[28] Qayum A., Rashid A., Liang Q., Wu Y., Cheng Y., Kang L., Liu Y., Zhou C., Hussain M., Ren X., Ashokkumar M., Ma H. (2023). Ultrasonic and homogenization: an overview of the preparation of an edible protein-polysaccharide complex emulsion. Compr. Rev. Food Sci. Food Saf..

[29] Ding Y., Liang Y., Luo F., Ouyang Q., Lin Q. (2020). Understanding the mechanism of ultrasonication regulated the digestibility properties of retrograded starch following vacuum freeze drying. Carbohydr. Polym..

[30] Raza H., Zhou Q., Cheng K.W., He J., Wang M. (2024). Synergistic impact of ultrasound-high pressure homogenization on the formation, structural properties, and slow digestion of the starch-phenolic acid complex. Food Chem..

[31] Ahmed Z., Chen J., Tufail T., Latif A., Arif M., Ullah R., Alqahtani A.S., Xu B. (2023). Fundamental opportunities and challenges of nutraceutical noodles enriched with agri-food by-products. Trends Food Sci. Technol..

[32] Tufail T., Ul Ain H.B., Ashraf J., Virk M.S., Ahmed Z., Dabbour M., Alsulami T., Althawab S., Xu B. (2025). Effect of triple-frequency sono-germination and soaking treatments on techno-functional characteristics of barley. Ultrason. Sonochem..

[33] Manzoor M.F., Waseem M., Diana T., Wang R., Ahmed Z., Mohamed Ahmed I.A., Ali M., An-Zeng X. (2025). Ultrasound-assisted modification to improve the red pepper seed protein isolate structural, functional, and antioxidant properties. Int. J. Biol. Macromol..

[34] Pan J., Zhang Z., Mintah B.K., Xu H., Dabbour M., Cheng Y., Dai C., He R., Ma H. (2022). Effects of nonthermal physical processing technologies on functional, structural properties and digestibility of food protein: a review. J. Food Process Eng.

[35] Karwasra B.L., Kaur M., Gill B.S. (2020). Impact of ultrasonication on functional and structural properties of Indian wheat (Triticum aestivum L.) cultivar starches. Int. J. Biol. Macromol..

[36] Golkar A., Milani J.M., Motamedzadegan A., Kenari R.E. (2022). Modification of corn starch by thermal-ultrasound treatment in presence of Arabic gum. Sci. Rep..

[37] Huang J., Wang Z., Fan L., Ma S. (2022). A review of wheat starch analyses: methods, techniques, structure and function. Int. J. Biol. Macromol..

[38] Ahmed Z., Xu B., Farooq U., Manzoor M.F., Awad M.F., Ashraf J., Tufail T., Abdi G. (2024). Impact of multi-frequency ultrasound processing with different treatment times on the structural quality of frozen wheat dough. Ultrason. Sonochem..

[39] Prasetyo T.A.B., Soegijono B. (2017). Effect of sonication process on natural zeolite at ferric chloride hexahydrate solution. J. Phys. Conf. Ser..

[40] Rahaman A., Kumari A., Zeng X.A., Adil Farooq M., Siddique R., Khalifa I., Siddeeg A., Ali M., Faisal Manzoor M. (2021). Ultrasound based modification and structural-functional analysis of corn and cassava starch. Ultrason. Sonochem..

[41] Han L., Cao S., Yu Y., Xu X., Cao X., Chen W. (2021). Modification in physicochemical, structural and digestive properties of pea starch during heat-moisture process assisted by pre- and post-treatment of ultrasound. Food Chem..

[42] Zhang K., Zhao D., Guo D., Tong X., Zhang Y., Wang L. (2021). Physicochemical and digestive properties of A- and B-type granules isolated from wheat starch as affected by microwave-ultrasound and toughening treatment. Int. J. Biol. Macromol..

[43] Basilio-Cortés U.A., González-Cruz L., Velazquez G., Teniente-Martínez G., Gómez-Aldapa C.A., Castro-Rosas J., Bernardino-Nicanor A. (2019). Effect of dual modification on the spectroscopic, calorimetric, viscosimetric and morphological characteristics of corn starch. Polymers.

[44] Chang R., Lu H., Bian X., Tian Y., Jin Z. (2020). Ultrasound assisted annealing production of resistant starches type 3 from fractionated debranched starch: structural characterization and in-vitro digestibility. Food Hydrocoll..

[45] Raza H., Ameer K., Ren X., Liang Q., Chen X., Chen H., Ma H. (2021). Physicochemical properties and digestion mechanism of starch-linoleic acid complex induced by multi-frequency power ultrasound. Food Chem..

[46] Yadav S., Mishra S., Vivek K., Mishra S. (2024). Characterisation of finger millet (Eleusine coracana) starch isolated by conventional and ultrasound-assisted starch isolation methods. Int. J. Food Sci. Technol..

[47] Flores-Silva P.C., Roldan-Cruz C.A., Chavez-Esquivel G., Vernon-Carter E.J., Bello-Perez L.A., Alvarez-Ramirez J. (2017). In vitro digestibility of ultrasound-treated corn starch. Starch - Stärke.

[48] Yang W., Kong X., Zheng Y., Sun W., Chen S., Liu D., Zhang H., Fang H., Tian J., Ye X. (2019). Controlled ultrasound treatments modify the morphology and physical properties of rice starch rather than the fine structure. Ultrason. Sonochem..

[49] He M., Chen L., Liu Y., Teng F., Li Y. (2025). Effect of ultrasonic pretreatment on physicochemical, thermal, and rheological properties of chemically modified corn starch. Food Chem..

[50] Zhang T., Guan E., Yang Y., Zhang L., Liu Y., Bian K. (2022). Underlying mechanism governing the influence of peanut oil addition on wheat dough viscoelasticity and Chinese steamed bread quality. LWT.

[51] Liu J., Qi Y., Hassane Hamadou A., Ahmed Z., Guo Q., Zhang J., Xu B. (2023). Effect of high-temperature drying at different moisture levels on texture of dried noodles: Insights into gluten aggregation and pore distribution. J. Cereal Sci..

[52] Hidalgo, B., Koporcic, M., Javier Cifuentes, J., Mujica, B., Castro, M., Videla, V., Quintero, M., Alarcon-Moyano, J., & Diaz-Calderon, P. (2023). Evaluation of pasting properties and retrogradation kinetic of a low viscosity potato starch, 03 July 2023, PREPRINT (Version 1) available at Research Square [ Doi:10.21203/rs.3.rs-3112247/v1].

[53] Scott G., Awika J.M. (2023). Effect of protein-starch interactions on starch retrogradation and implications for food product quality. Compr. Rev. Food Sci. Food Saf..

[54] Chakraborty I., Govindaraju I., Kunnel S., Managuli V., Mazumder N. (2023). Effect of storage time and temperature on digestibility, thermal, and rheological properties of retrograded rice. Gels (basel, Switzerland).

[55] Woodbury T.J., Pitts S.L., Pilch A.M., Smith P., Mauer L.J. (2023). Mechanisms of the different effects of sucrose, glucose, fructose, and a glucose-fructose mixture on wheat starch gelatinization, pasting, and retrogradation. J. Food Sci..

[56] Shi M., Wang F., Lan P., Zhang Y., Zhang M., Yan Y., Liu Y. (2021). Effect of ultrasonic intensity on structure and properties of wheat starch-monoglyceride complex and its influence on quality of norther-style chinese steamed bread. LWT.

[57] Ding Y., Xiao Y., Ouyang Q., Luo F., Lin Q. (2020). Modulating the in vitro digestibility of chemically modified starch ingredient by a non-thermal processing technology of ultrasonic treatment. Ultrason. Sonochem..

[58] Kaur H., Gill B.S. (2019). Effect of high-intensity ultrasound treatment on nutritional, rheological and structural properties of starches obtained from different cereals. Int. J. Biol. Macromol..

[59] Yadav P., Bharathi U., Suruthi K., Bosco S.J.D. (2024). Effect of ultrasonic modification on physiochemical, structural, functional properties and *in vitro* starch digestibility of *Amaranthus paniculatus* (Rajgeera) starch. Biomass Convers. Biorefin..

