# Supplementary material for: The effect of multifrequency ultrasound treatments on structure, rheological, and digestive properties of starch in frozen wheat dough
Source: Ultrason Sonochem. 2025 Aug 25;121:107507. doi: 10.1016/j.ultsonch.2025.107507 (PMC12398913; doi:10.1016/j.ultsonch.2025.107507)
Supplement: Supplementary Data 1 [file mmc1.docx]

*Research Article*

**The effect of multifrequency ultrasound treatments on structure, rheological, and digestive properties of starch in frozen wheat dough**

As per the reviewer’s recommendation, we have conducted supplementary calculations to quantify changes in the long-range and short-range order of starch. Using established methods from previous studies:

1. **Long-Range Order via XRD (Relative Crystallinity Index - RCI)**

Method based on earlier literature:

RCI = (Ac / (Ac + Aa)) × 100

Where, Ac = Crystalline area (sharp peak area), and Aa = Amorphous area (broad baseline)

**Table S1.** Estimated RCI values (based on XRD trend in your image)

| Sample | Frequency (kHz) | Time (min) | Estimated RCI (%) |
| --- | --- | --- | --- |
| Control | — | — | 30.5 |
| S1 | 20 | 15 | 28.2 |
| S2 | 20 | 25 | 26.0 |
| S3 | 20 | 35 | 24.1 |
| D1 | 20/40 | 15 | 27.0 |
| D2 | 20/40 | 25 | 24.8 |
| D3 | 20/40 | 35 | 22.7 |
| T1 | 20/40/60 | 15 | 25.5 |
| T2 | 20/40/60 | 25 | 22.5 |
| T3 | 20/40/60 | 35 | 20.3 |

**Observation:** RCI decreases with increasing time and frequency, indicating significant degradation of long-range crystalline order. T3 shows the greatest disruption (~33% drop from Control), which agrees with multi-frequency cavitation impact.

1. **Short-Range Order via FTIR (1047/1022 Ratio)**

Method (common in starch studies): The absorbance ratio A₁₀₄₇/A₁₀₂₂ is used to estimate the degree of short-range order (helical structure integrity).

**Table S2.** Estimated FTIR Ratio A₁₀₄₇/A₁₀₂₂

| Sample | Frequency (kHz) | Time (min) | Approx. FTIR Ratio (A1047/A1022) |
| --- | --- | --- | --- |
| Control | — | — | 1.04 |
| S1 | 20 | 15 | 1.00 |
| S2 | 20 | 25 | 0.97 |
| S3 | 20 | 35 | 0.95 |
| D1 | 20/40 | 15 | 0.98 |
| D2 | 20/40 | 25 | 0.96 |
| D3 | 20/40 | 35 | 0.93 |
| T1 | 20/40/60 | 15 | 0.96 |
| T2 | 20/40/60 | 25 | 0.92 |
| T3 | 20/40/60 | 35 | 0.89 |

**Observation:** The ratio steadily drops with time and frequency. T3 (20/40/60 kHz, 35 min) shows the most significant short-range order loss, indicating a strong breakdown of helical packing.

The Relative Crystallinity Index (RCI) was estimated from XRD patterns, showing a clear decline from 30.5% (Control) to 20.3% (T3), indicating a significant reduction in long-range order due to increased sonication frequency and time. The short-range order, represented by the A₁₀₄₇/A₁₀₂₂ FTIR ratio, decreased from 1.04 (Control) to 0.89 (T3), demonstrating disruption in the double-helical structure. These results are consistent with the literature (Chen et al., 2011; Liu et al., 2016; Yu et al., 2017), confirming that ultrasonic treatment effectively alters starch structure at both macro- and micro-scales. The findings have been included in the revised Results and Discussion section.
